# Early Intervention with a Compression Sleeve in Mild Breast Cancer-Related Arm Lymphedema: A 12-Month Prospective Observational Study

**DOI:** 10.3390/cancers15102674

**Published:** 2023-05-09

**Authors:** Karin Johansson, Katarina Blom, Lena Nilsson-Wikmar, Christina Brogårdh

**Affiliations:** 1 Department of Health Sciences, Lund University, 221 00 Lund, Sweden; 2 Physiotherapy Cancer, Karolinska University Hospital, 171 76 Stockholm, Sweden; 3 Department of Neurobiology, Care Sciences and Society, Karolinska Institutet, 141 52 Huddinge, Sweden; 4 Department of Neurology, Rehabilitation Medicine, Memory Disorders and Geriatrics, Skåne University Hospital, 221 85 Lund, Sweden

**Keywords:** breast cancer, lymphedema, prevention, treatment, clinical research

## Abstract

**Simple Summary:**

Chronic lymphedema in the arm is a rather common side-effect of breast cancer treatment and its prevention is desired. The most important and evidence-based treatment of arm lymphedema is daily use of a compression sleeve. In our previous randomized controlled trial, it was shown that early treatment with a compression sleeve in compression class 1 in mild arm lymphedema can prevent progression for 6 months. The aim of the present study was to follow the progression/no progression for 12 months. It was revealed that the results from the 6-month intervention were persistent at 9- and 12-months follow-up. Therefore, it can be concluded that a compression sleeve (compression class 1) may be applied immediately after early diagnosis of lymphedema to prevent progression and to avoid mild arm lymphedema becoming chronic.

**Abstract:**

Background: In our previous randomized controlled trial (RCT), the progression/no progression of mild breast cancer-related arm lymphedema (BCRL) was examined among women randomized to a compression group (CG) with a compression sleeve (compression class (ccl) 1) or not (NCG) for 6 months. In the present prospective study, BCRL in the CG and NCG was followed for 12 months. Methods: At the end of the RCT, 33 women with mild BCRL were eligible in the CG and 37 in the NCG. The proportional differences in no progression/progression of BCRL were defined as a >2% increase from start of RCT or exceeding 10% in the lymphedema relative volume as measured by the water displacement method. In addition, changes in the lymphedema relative volume and tissue dielectric constant ratio, which measures local tissue water, were examined. At the end of the RCT (i.e., after 6 months), a one-month break of the compression treatment was made in the CG. If the lymphedema relative volume progressed by definition, the compression treatment was resumed and continued, with follow-up of all women at 9 and 12 months. Results: A larger proportion of women in the NCG showed progression (57%, 61%, 67%) compared to the CG (16%, 22%, 31%) at 6, 9, and 12 months (*p* < 0.001, 0.005, 0.012), respectively. Twelve (33%) women in the NCG did not progress at all. No changes of the lymphedema relative volume and local tissue water were found over time at any follow-ups, but were stable on a low level. Conclusions: To avoid the progression of mild BCRL into a chronic issue in the long-term, compression sleeve ccl 1 may be applied immediately after early diagnosis of mild BCRL.

## 1. Introduction

During the last few decades, the survival rate of cancer has improved, but has also left an increasing number of patients suffering from side-effects from cancer treatments, such as lymphedema. Breast cancer-related lymphedema (BCRL) is characterized by a swelling of the arm, hand, or breast, or at the trunk close the axilla. Although non-invasive surgery is increasingly performed for breast cancer and provides fewer morbidities [1], the risk factors for BCRL include more extensive surgery (axillary lymph node dissection, greater number of lymph nodes dissected, mastectomy), radiotherapy, and a higher body mass index (BMI) [2]. Other potential risk factors are chemotherapy, a sedentary lifestyle [2], and a genetic predisposition [3]. The reported incidence of BCRL varies depending on the definitions and measuring methods used. In a cross-sectional study, Johansson & Branje [4] identified women with BCRL in a surveillance program of breast cancer patients with axillary dissection and found an incidence of 38.7% over a 10 year-period, whereas Kilgore et al. found an incidence of 34% over 2 years [5]. The standard treatment of mild arm lymphedema encompasses wearing a compression garment during the daytime and providing information about self-care, such as exercise, weight control, skin care, and self-massage. Of these components, compression therapy is shown to be the most important to reduce the lymphedema volume [6].

Lymphedema is considered to be a chronic condition and progression can be expected without treatment [7]. However, even with treatment, some mild cases may develop severe lymphedema [8]. Several studies have shown that early diagnosis and treatment is important to prevent progression [9,10]. However, a recent RCT including 143 patients with mild BCRL defined by a relative arm volume increase (RAVI) failed to show a preventive effect of a 1-year compression treatment at a 2-year follow-up [11]. Some studies indicate that it is the edema volume at the start of treatment that is the most important predictive factor for the treatment outcome [4,12,13]. However, in mild BCRL, the lymphedema can be local [14] or segmental [15]. When lymphedema starts, it is local because of dermal backflow [16], which develops due to lymphatic obstruction. When pressure rises in the vessels, the fluid flows backwards into the dermal lymphatic capillaries [17]. The lymph fluid then leaks out into the dermal tissue. The use of a tissue dielectric constant makes it possible to identify local lymphedema earlier [16] as compared to the lymphedema relative volume in 45% of patients at risk for BCRL [18]. Therefore, intervention can start earlier, regardless of the initial lymphedema volume. In our recent RCT [19], it was revealed that the proportion of patients with progression was much smaller for the group using compression sleeves for 6 months (16%), compared to the controls wearing no compression (57%). Currently, there is limited knowledge about the long-term effect of early treatment with a compression sleeve. Therefore, the aim of the present study was to examine (i) the proportional difference in progression/no progression of arm lymphedema in mild BCRL and (ii) the changes in arm volume and local tissue water at 9- and 12-months follow-up, when treated with compression sleeve or not for 6 months.

## 2. Method

### 2.1. Study Design

This is a prospective, observational, multicentre study of 9- and 12-months follow-up in women with mild BCRL that were randomized to use a compression sleeve or not for 6 months. 

### 2.2. Participants

Seventy-five women treated for unilateral breast cancer with axillary node dissection and diagnosed with mild arm lymphedema at the Lymphedema Unit, Skåne University Hospital and at the Physiotherapy Cancer Unit, Karolinska University Hospital, Sweden, had been included in our prior RCT. The women were excluded if they had recurrent cancer, concurrent diseases, cognitive impairments, or were unable to speak or understand the Swedish language [19].

### 2.3. Ethical Approval

The study was approved by the Regional Ethical Board, Lund University, Sweden, Dnr: 2014/399. All the participants provided informed consent and the data were collected from September 2014 to October 2019.

### 2.4. Procedures 

The women had been called for routine clinical follow-up visits in a surveillance program [19,20]. Mild arm lymphedema was defined as palpated increased skin and subcutis thickness of the affected arm compared with the non-affected arm [4,18,21], in addition to either a threshold tissue dielectric constant ratio (≥1.45 in the upper arm and/or ≥1.3 in the forearm) [22] and/or lymphedema relative volume ≥ 5 to ≤8% [23,24]. When mild arm lymphedema was diagnosed, the women were included in the RCT [19].

### 2.5. Randomization

The 75 women with mild BCRL were randomized to treatment for 6 months with compression or not, referred to as the compression group (CG) or the non-compression group (NCG). The procedure is explained more in detail elsewhere [19].

### 2.6. Description of the Interventions

All the patients were given routine educational strategies, including brief information of the function of the peripheral lymph system and impairments caused by surgery and irradiation, as well as advice about self-care, including exercise, weight control, skin care, and self-massage. The information provided about exercise was based on recommendations for cancer survivors with the purpose of reducing the risk for recurrence of cancer [25,26]. To avoid weight gain and skin infections, which can potentially worsen lymphedema, advice about weight control and skin care were also given [27,28]. The education was given both orally at a face-to-face session and written in a leaflet. 

To perform self-massage, the women were taught to apply light strokes to the shoulder and arm for about 10–15 min a day with the intention to reduce their symptoms, such as a feeling of tightness and pain, based on the gate control theory [29]. If the self-massage was perceived as effective, the participants were encouraged to continue, and if not, they stopped [19]. The CG also received circular knit compression sleeves in compression class (ccl) 1 or, if needed, individually adjusted compression sleeves (ccl 2) for daily wearing for six months. Thereafter, the wearing of the compression sleeve was reduced to half a day for two weeks, and then no sleeve for another two weeks. If the lymphedema volume at this timepoint had increased in the lymphedema relative volume ≥ 2% from start of the RCT, the compression treatment was resumed and continued. During the RCT or follow-up, if the women in the NCG increased in the lymphedema relative volume ≥ 2%, they began to wear a compression sleeve. The threshold of 2% was chosen as the minimal detectible change of lymphedema and is shown to be 1.0%, as measured by the water displacement method [30,31]. If the lymphedema relative volume exceeded ≥ 10% for any woman in any group, they were excluded from the study and received extended treatment. The thresholds of 5–10% for minimal lymphedema is recommended by the International Society of Lymphology [32].

### 2.7. Background Data 

At the start of the RCT, data of surgical and adjuvant treatments were retrieved from the participants’ medical records, and their BMI was calculated and recorded. The women responded to a study-specific questionnaire with questions regarding age, education level, and marital status. 

### 2.8. Primary Outcome 

The primary outcome was the proportional difference between the CG and NCG showing progression/no progression (increase in the lymphedema relative volume ≥ 2% from start of the RCT or exceeding ≥10%) at end of the 6-month RCT, and 9 and 12 months after start of the RCT.

### 2.9. Secondary Outcomes

The secondary outcomes were the differences between the CG and NCG in terms of the changes of the lymphedema relative volume and tissue dielectric constant ratio, from the start of the RCT until the end of the 6-month RCT, and at 9 and 12 months.

### 2.10. Measurements

The arm volume and local tissue water were measured at the start and end of the RCT, and at 9 and 12 months. Self-care, including weight control, the use of a compression garment, frequency of self-massage, and level of physical activity/exercise, was monitored during the 12 months.

#### 2.10.1. Arm Volume

The arm volume was measured with the1 water displacement method by lowering the arm into a container filled with water with the elbow extended and the hand placed on the bottom of the container. The lymphedema relative volume was obtained by calculating the percentage difference in the lymphedema volume between the affected and non-affected arm. The measurements of the lymphedema relative volume were adjusted by −1.5% if present in the dominant arm and +1.5% if present in the non-dominant arm [33]. The water displacement method has been shown to be reliable (intraclass correlations of 0.99) with small measurement errors (SEM of 0.1%) [30,31]. 

#### 2.10.2. Local Tissue Water

The local tissue water was evaluated by the MoistureMeterD and MoistureMeterD compact (MMDC) (Delfin Technologies Ltd., Kuopio, Finland). A low-intensity 300MHz signal is transmitted from the probe in contact with the skin. Based on information from the reflected wave, a tissue dielectric constant value is calculated. The probe has a penetration depth of 2.5 mm and corresponds to a maximum of 78.5% of pure water content based on the percentage of water content in the body. The presented tissue dielectric constant ratios are approximately the same as the new device MMDC, corresponding to a maximum of 100% pure water content based on a 100% scale [34]. Each site was measured once [35] at six points: 5 cm proximal and 5 cm distal to the antecubital fossa (medial, frontal, and lateral). If lymphedema was palpated more proximally or distally in the arm, complementary measurements were made 15 cm proximal or distal to the antecubital fossa. A significant positive correlation between arm tissue dielectric constant ratios and arm volume (*p* < 0.001, *r* = 0.690) has been reported [36].

#### 2.10.3. Self-Care

At 12-months follow-up, the body weight was noted. Additionally, the use of a compression garment was rated on a three-point scale (not at all, half the day, or the whole day) the frequency of performed self-massage was rated on a four-point scale (no massage, seldom, two-three times a week, or every day) and the physical activity level/exercise during the last four weeks was rated by the participants on a six-point scale (from sedentary to high physical activity) [37]. 

### 2.11. Statistical Power and Analysis

The power calculation for the RCT (a power of 0.85 at *p* ≤ 0.05 level of significance) showed that 80 participants should be included. In the present study, descriptive statistics are presented as mean ± SD for continuous values and as a number and proportion for categorical variables. The differences between the groups were calculated with a *t*-test for continuous data, and Fisher–Freeman–Halton exact test or Pearson Chi square test for nominal data. The tissue dielectric constant data (not normally distributed) are presented as both median (min–max) and mean ± SD, and the differences between the groups were calculated with Mann–Whitney’s test. The women who exceeded a lymphedema relative volume ≥ 10% were not included in the analyses of lymphedema relative volume and tissue dielectric constant ratio after the timepoint of exceedance. Statistical analyses were carried out in IBM SPSS Statistics 28 and a significance level of *p* < 0.05 (two-tailed) was chosen.

## 3. Results

**Figure 1:**
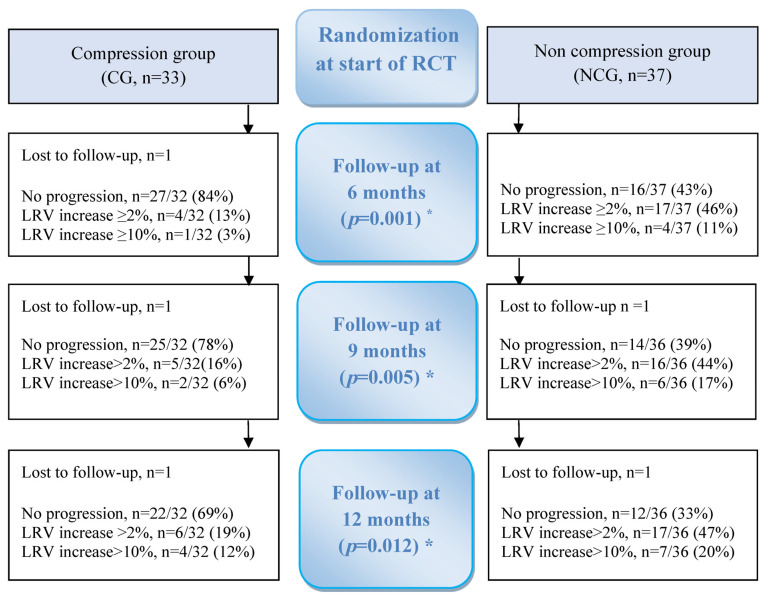
Flowchart of randomization to compression group (CG) or no compression group (NCG) and primary outcome being the proportional difference between the groups, showing progression/no progression of mild breast cancer-related lymphedema for a 6-month intervention and at follow-ups. No progression = Lymphedema relative volume did not increase ≥2% compared to start of RCT or exceed ≥10%. LRV = lymphedema relative volume, including all women showing progress by an increase ≥2% compared to start of RCT or exceeding ≥10%. * Indicates differences between groups, calculated by Fisher–Freeman–Halton exact test or Pearson Chi square test.

At the start of the RCT, 75 women were included. However, during the trial, five women were excluded mainly due to a recurrence of the cancer disease, and 70 were finally randomized to the CG or NCG. One woman was lost to follow-up during the RCT, thus 69 women were available for data analysis at 6 months: 32 in the CG and 37 in the NCG (Figure 1).

### 3.1. Differences between the Groups at Start of the RCT and at 12-Months Follow-Up

The basic data of the groups are presented in Table 1. At start of the RCT, the women in the CG and NCG were comparable in data except for surgery. In the CG, 61% had surgery with mastectomy, compared to 35% in the NCG (*p* = 0.033) (Table 1). At the start of the RCT, the lymphedema relative volume in the CG/NCG were, on average, 4.4 ± 3.1%/3.8 ± 3.5% (Table 2), and the highest tissue dielectric constant ratios were 1.53 ± 0.21/1.51 ± 0.30, respectively (Table 3), with no differences between the groups. No changes in BMI were found from start of the RCT (mean (SD) in the CG 26.1(4.8)/NCG 27.2(5.4)) to 12-months follow-up (CG 26.1(4.6)/NCG 26.4(5.2)), and there were no differences between the groups concerning physical activity/exercise or frequency of self-massage.

### 3.2. Use of Compression Sleeves

At the start of the RCT, all the women in the CG were treated with ready-made compression sleeves (ccl 1), except four women who received individually adjusted sleeves (ccl 2), and all received two pieces each. In nine women, the hand was also affected, and a compression glove was prescribed. After 6 months, 92% of women in the CG stated that they used the sleeve the whole day, and 8% half the day. After the end of the RCT, the CG stopped the compression treatment and 22 (69%) showed no increase of the lymphedema relative volume (≥2%) or exceeded ≥10% and continued without compression to the 12-month follow-up. The corresponding number in the NCG was 12 (33%) (Figure 1). At 12-months follow-up, four women in each group used arm sleeve ccl 2, and five in the NCG and six in the CG used gloves.

### 3.3. Primary Outcome

#### Proportion of Progression/No Progression of BCRL for 12 Months 

In Figure 1, the proportion of women with progression/no progression of BCRL for 12 months is presented. A significantly larger proportion in the NCG than in the CG shows progression of BCRL at all follow-ups compared to the start of the RCT. At 6 months from the start of the RCT, 5/32 (16%) in the CG showed progression, compared to 21/37 (57%) in the NCG (*p* = 0.001). At 9 months from the start of the RCT, 7/32 (22%) in the CG showed progression, compared to 22/36 (61%) in the NCG (*p* = 0.005). At 12 months, 10/32 (31%) in the CG showed progress compared to 24/36 (67%) in the NCG (*p* = 0.012). 

### 3.4. Secondary Outcomes

#### 3.4.1. Lymphedema Relative Volume for 12 Months

At the start of the RCT, there was no difference between the groups in the lymphedema relative volume. At 6 months, the CG had a lower lymphedema relative volume compared to the NCG (*p* = 0.004), but not at 9 and 12 months.

At 6 months, compared to the start of the RCT, the mean lymphedema relative volume decreased −3.8% in the CG, and increased +0.1% in the NCG (*p* < 0.001). No differences between the groups were found at 9 and 12 months (Table 2). 

#### 3.4.2. Changes in Local Tissue Water for 12 Months

The measurement point for the highest ratio at the start of the RCT was followed, and no differences were found between the CG and the NCG at any time point (Table 3).

## 4. Discussion

To the best of our knowledge, this is the first study evaluating the long-term results from a 6-month RCT including women with early and mild BCRL based on the evaluation of two properties of lymphedema, namely local tissue water and volume change. The study aimed to investigate the proportion of women showing progression/no progression of BCRL after treatment with compression garments or not. We found that the results from the RCT concerning the differences in proportions between the groups, which favor the CG, were persistent at the 9- and 12-month follow-ups.

The proportions of women with progress were significantly larger at 9- and 12-months follow-ups in the NCG compared to the CG. A better result for the CG was found despite a greater number of women with mastectomy in the CG, which is considered a risk factor for lymphedema. In addition, most of the women in the CG stopped wearing compression after 6 months of treatment (i.e., at the end of the RCT), which might have had a potential negative influence on lymphedema.

This may suggest that early compression treatment is beneficial compared to delayed treatment, that is, to start with compression immediately when lymphedema is detected and not only when deterioration is found. These results are consistent with the findings in the study by Akita et al., who diagnosed early BCRL based on the classification of different lymph flow patterns with indocyanine green lymphography and started compression therapy. An improvement in lymphatic function, presented as change to a less severe class or into the normal class, was confirmed at 5.0 ± 2.1 months after onset in 31% of the patients, and did not recur during 10.4 ± 5.4 months [38]. The study was not an RCT, but the results indicate that compression therapy may decrease fluid filtration into the tissue [39], thereby reducing the burden on the lymphatic system, which may support faster recovery compared to no compression treatment. 

On the other hand, as no controls were included in the study by Akita et al. [38], the findings may also show a normal procedure of restoration of the lymphatics. This may explain why 33% in the NCG in the present study showed no progression during 12-months of follow-up. The finding is in line with results from a study using multi-frequency bioimpedance spectroscopy (BIS) L-Dex to measure mild BCRL, which demonstrated that an increase in BIS at 6 months did not predict lymphedema at 24 months [13]. Further research is needed to investigate normal regeneration, as well as the impact of compression treatment for prevention and early treatment of BCRL.

The important finding that 33% of the women in the NCG showed no progression must be considered in all research on the prevention and treatment of early mild lymphedema. Regression of lymphedema, particularly in the first year after surgery, has also been noted by others. Kilbreath et al. diagnosed BCRL with BIS and found that some BCRL lymphedema was transient and only 23% of participants still had lymphedema 15 months after surgery [40], which left 77% without lymphedema, compared to 33% at 12 months in the present study. However, the BIS method has been found to be less sensitive than the tissue dielectric constant applied in the present study, and may therefore show false negative values [21], which may explain the larger proportion of no lymphedema.

Others have obtained good results of early treatment of mild BCRL, but with only one-month intervention and no long-term follow-up [41]. However, in our 6-month RCT evaluating early treatment with a compression sleeve [19], the largest improvement occurred after the first month, but with a continuing decrease of the lymphedema relative volume from mean 4.4% at the start to 0.7% after 6 months (Table 2). Furthermore, in a retrospective study, Johansson and Branje [4] included 70 women with BCRL, treated them with a compression sleeve, and found a decrease in the lymphedema relative volume from a mean 8.6% at the start to 7.3% after 12 months. Therefore, it is likely that the largest effect is found during the first months of compression treatment. Additionally, in the present study, only 15% (from 16% at 6 months to 31% at 12 months; see Figure 1) in the CG showed progression after having stopped wearing the compression sleeve. It is therefore reasonable to believe that the treatment period should be about 6 months and will be enough in most cases. However, the optimal duration is most likely individual, depending on the local conditions, in particular the disturbance of the lymph flow with more or less spread of dermal backflow in the subcutaneous tissue [17]. The most relevant clinical care must therefore include applying compression for 6 months, then a stepwise reduction of the use of compression to determine who should continue with compression and who can be without.

Although the present study shows that wearing a compression sleeve has an impact on the progression of BCRL, a recently published larger (n = 143) study failed to demonstrate the same [11]. An important difference between the two studies may be that only the arm volume was used for the diagnosis of mild BCRL. We know by now that both the tissue dielectric constant ratio and the lymphedema relative volume must be applied, as they measure different aspects of edema [21]. A measurement of the local tissue water can determine the diagnosis at a very early stage, earlier than by an arm volume measurement alone [19]. This very early diagnosis may have increased the potential to influence, for example, the dermal backflow, as discussed above.

The water displacement method is considered to be the “gold standard” in arm lymphedema measurements [42]. However, calculation of the limb volume using circumference measurements every 4th or 8th centimeter along the limb [43] is another reliable method for extremity volume measurements. It is probably the most widely used method, as it requires cheaper equipment, but it is more time-consuming than the water displacement method. Concerning the tissue dielectric constant measurements, evaluating the local tissue water by lymphfluoroscopy can detect local lymphedema in the subcutaneous tissue. However, lymphfluoroscopy cannot quantify lymphedema, but rather determines the stages [17], and therefore cannot be used easily for the evaluation of treatment. The same goes for palpation of increased subcutaneous tissue compared to the healthy side. Palpation can be applied for the detection of early lymphedema [4,16], but it cannot be used for the evaluation of treatment, as it is not, in practice, possible to palpate small changes.

The women were educated in self-care, including information about exercise, weight control, skin care, and self-massage. It can be argued that education may influence lymphedema. However, at 12-months follow-up, there were no differences between the groups concerning their exercise level, BMI, or self-massage. Regarding exercise, it should also be noted that the lymph system works differently compared to the blood system by pumping fluid on its own through a great number of lymphangions [39]. The lymph system may therefore also react differently due to exercise and need another kind of approach. This is maybe why a recent meta-analysis of exercise for the treatment of cancer-related lymphedema including more than 600 participants failed to show any improvement in lymphedema [44]. Another part of the self-care program is self-massage with light strokes over the arm and shoulder for the purpose of decreasing symptoms, such as pain and tightness, based on the gate effect theory. A special massage method to stimulate lymph drainage with light massage using certain grips was developed especially for the lymph system and has been used as a treatment for many years. However, it has been shown in several meta-analyses that manual lymph drainage has no effect on lymphedema [6,45,46], even when it is guided by lymphflouroscopy, which highlights where the lymph flow could be increased by massage [47]. Therefore, we believe that neither exercise nor light massage had the potential to influence lymphedema in the present study. 

The fact that no differences between the groups were found in relatively small-volume lymphedema at 9 and 12 months, and that the mean lymphedema relative volume was small, may have several explanations. Firstly, most women in the CG stopped wearing the compression sleeve after 6 months (the end of the RCT) and did not progress, but maintained lower levels of lymphedema. Secondly, the women in the CG that progressed after one month of no compression started to wear the sleeve again, stopping the progression. The entire CG showed no progress compared to values found before the start of the RCT (4.4% to 4.2%). Thirdly, the women in the NCG who received compression treatment if they increased >2% in the lymphedema relative volume (i.e., a delayed treatment) showed no progression after the delay compared to the start of the RCT (2.9% to 3.0%). Fourthly, the rest of the NCG did not progress, but were stable. The entire NCG showed no progression from the reported values before start of the RCT. However, it must be taken into account that more women in the NCG exceeded the lymphedema relative volume of 10% and dropped out. Nevertheless, this procedure shows that all the women that progressed within the lymphedema relative volume of 10% and needed compression received sufficient treatment, and those who did not progress could continue without compression, whether they belonged to the CG or to the NCG from the start. 

The International Lymphedema Framework recommends the use of low compression (14–18 mmHg) for mild upper limb lymphedema with no shape distortion [48]. In the present study, most of the women with mild BCRL were prescribed a round knit arm sleeve in ccl 1 (15–21 mm Hg). During the RCT, the compression treatment resulted in a significant decrease in the lymphedema relative volume and the adherence to wearing the compression sleeve was good, indicating that a ccl 1 sleeve is sufficiently effective and well-tolerated [19].

Although the present study showed that 69% of the women in the CG could discontinue wearing the compression sleeve after 6 months and 33% of the women in the NCG did not need compression at all, compression treatment is the most important of all conservative lymphedema treatment methods [6]. An optimal compression garment and compliance are very important to improve patient outcomes. However, it can be a challenge for the lymphedema therapist to motivate patients with mild arm lymphedema to use compression for a longer time when they have no or minor experiences with symptoms in the arm. In a qualitative study of 16 women receiving lymphedema treatment, it was revealed that some women stopped using the compression garment by their own initiative due to problems with the arm sleeve or because of stabilization of the lymphedema. In addition, the appearance of the limb and comments from others reduced the patients’ motivation to use a compression garment [49]. Considering these results, it may be easier to motivate the patient to use compression only for 6 months to begin with, and then evaluate whether it is needed further or not. 

At the 12-month follow-up, 12% of the women in the CG had exceeded the lymphedema relative volume of ≥10% compared to 20% in the NCG. We consider these women to have irreversible lymphedema. In a 5-year follow-up of mild BCRL by Bar Ad et al., it was found that 21% progressed after 4 years or longer [8], meaning that progression can occur even after several years in a stable condition. We found that women in the present study progressed over time. A total of 37% women exceeded the lymphedema relative volume ≥ 2% from the start of the RTC or lymphedema relative volume ≥ 10%, and of these, 19% wore compression arm sleeves. Bundred et al. also found that progression to moderate lymphoedema occurred in 15% patients even after sleeve application [13]. Zaleska et al. [50] used indocyanine green near-infrared lymphangiography to investigate the effect of treatment with bandaging. They found that bandaging was effective in moving edema fluid, but in some cases, no improvement was seen despite applying high compression force with the bandaging [50]. The non-responsiveness to compression treatment can perhaps be explained by individual variation in dermal backflow and the restoration of lymphatic pathways after surgery. Suami et al. examined patients after axillary surgery with lymphangiography and indocyanine green fluorescence lymphography and found that different patterns of lymphatic drainage may determine the severity of lymphedema [51].

The risk that progression may occur in mild BCRL after a longer period of no compression calls for providing information to women about signs and self-measurements so that they can detect progression themselves. For the group of women that progress despite using compression, further monitoring is necessary, including proper treatment with optimal compression and self-care. They must be carefully followed by objective measurements until progression is stopped, and, in the best-case scenario, the lymphedema volume is reduced.

## 5. Strength and Limitations

The strength of the study is that we have gained more knowledge about the benefit of compression/no compression on BCRL over a longer perspective. Additionally, the groups were well-matched and there was a high level of compliance regarding wearing arm sleeves. The measurements with the water displacement method and tissue dielectric constant were coordinated and almost all the measurements were performed by the same two experienced lymphedema therapists, with few missing data. The power analysis for the RCT revealed that 80 participants should be included. Finally, 75 were included due to limit of time, and 68 (CG, n = 32/NCG, n = 36) were eligible for analysis at the 12-month follow-up. However, as there were significant results with low p-values for the primary outcome at all follow-ups, we consider the power to be acceptable. Another limitation was related to ethical considerations. Because some women in the NCG had to start treatment (47%) but were included in analysis, and some were excluded after the time-point when the lymphedema relative volume exceeded ≥ 10% (20%), in the results may be insignificant. However, for ethical reasons, we had to undertake these procedures in order to avoid risking any further progression of the lymphedema.

## 6. Conclusions

After 6 months of treatment with a compression sleeve or not, a significantly larger proportion of women in the NCG showed progression of mild BCRL compared to the CG at the 9- and 12-month follow-ups. However, more than 30% of the NGC did not progress at all. No changes of the lymphedema relative volume and local tissue water were found at any follow-ups, but were stable at a low level. To avoid the progression of mild BCRL into a chronic condition, a compression sleeve ccl 1 may be applied immediately after early diagnosis, when the BCRL still is mild. 

## Figures and Tables

**Table 1:** Basic data for women with mild breast cancer-related arm lymphedema (n = 70) at start of randomized controlled trial, in compression group (CG), and non-compression group (NCG).

	CG n = 33	NCG n = 37	*p*-Value *
Age in years at diagnosis, mean (SD)	57.9(13.8)	57.0(12.5)	0.795
BMI kg/m^2^, mean (SD)	26.1(4.8)	27.2(5.4)	0.390
Surgery			**0.033**
Mastectomy and ALND, n (%)	20(61)	13(35)	
Lumpectomy and ALND, n (%)	13(39)	24(65)	
Lymph nodes			
Removed at surgery, mean (SD)	14.9(5.8)	16.0(5.5)	0.397
With metastasis, mean (SD)	2.3(3.0)	2.6(2.4)	0.655
Adjuvant treatment			
Radiotherapy, n (%)	32(97)	37(100)	0.471
Chemotherapy, n (%)	27(82)	33(89)	0.499
Hormone therapy, n (%)	25(76)	28(76)	0.994
Affected side			
Right/left, n	18/15	19/18	0.789
Dominant ^a^, n (%)	19(58)	17(47)	0.390
Lymphedema			
Time from surgery to onset, months, mean (SD)	6.1(5.5)	6.6(5.2)	0.721
Duration, months, mean (SD)	1.0(1.3)	1.0(1.5)	0.834
In hand, self-rated ^a^, n (%)	9(27)	9(25)	0.830

ALND: axillary node dissection. * Significance level 0.05, *t*-test for continuous data, Pearson Chi-square test or Fisher’s exact test for nominal data. ^a^ Missing value: 1.

**Table 2:** Lymphedema relative volume (LRV) in women with mild breast cancer-related arm lymphedema at 6-, 9-, and 12-months follow-up after being randomized to compression group (CG) or non-compression group (NCG), (n = 70).

LRV % ^a^	CG	NCG	*p*-Value *
**At start of RCT**	n = 33	n = 37	
Mean ± SD	4.4 ± 3.1	3.8 ± 3.5	0.456
At 6 months	n = 30	n = 33	
Mean ± SD	0.7 ± 3.0	3.6 ± 4.5	**0.004**
Change from start			
Mean diff (CI)	−3.8 (−5.0 to −2.5)	0.1 (−1.1 to 1.2)	**<0.001**
**At 9 months**	n = 30	n = 28	
Mean ± SD	3.2 (3.3)	3.5 (5.0)	0.799
Change from start			
Mean diff (CI)	−1.3 (−2.7 to 0.1)	0.2 (−1.3 to 1.7)	0.143
**At 12 months**	n = 30	n = 25	
Mean ± SD	4.2 (3.6)	2.6 (4.2)	0.143
Change from start			
Mean diff (CI)	−0.3 (−1.8 to 1.1)	−0.4 (−1.9 to 1.0)	0.900

* Significance level 0.05, independent *t*-test. ^a^ Adjusted with +1.5%, if surgery in non-dominant side and −1.5% if surgery in dominant side. Women were not included in analysis after the time-point when LRV exceeded ≥ 10%.

**Table 3:** Tissue dielectric constant (TDC) ratio in women with mild breast cancer-related arm lymphedema at 6-, 9-, and 12-months follow-up after being randomized to compression group (CG) or non-compression group (NCG), (n = 70).

TDC ^a^	CG	NCG	*p*-Value *
**At start of RCT**	n = 33	n = 37	
Median (min–max)	1.50 (1.09 to 1.96)	1.55 (1.05 to 2.15)	0.805
Mean ± SD	1.53 ± 0.21	1.51 ± 0.30	
**At 6 months**	n = 29	n = 31	
Median (min–max)	1.12 (0.77 to 1.84)	1.18 (0.91 to 1.90)	0.689
Mean ± SD	1.23 ± 0.24	1.28 ± 0.29	
Change from start			
Median diff (min–max)	−0.28 (−1.04 to 0.30)	−0.16 (−1.00 to 0.40)	0.375
Mean diff (CI)	−0.29 (−0.39 to −0.20)	−0.25 (−0.36 to −0.13)	
**At 9 months**	n = 28	n = 27	
Median (min–max)	1.18 (0.73 to 2.14)	1.24 (0.97 to 1.74)	0.266
Mean ± SD	1.21 ± 0.29	1.26 ± 0.22	
Change from start			
Median diff (min–max)	−0.32 (−0.91 to 0.29)	−0.21 (−1.15 to 0.50)	0.567
Mean diff (CI)	−0.31 (−0.42 to −0.20)	−0.27 (−0.40 to −0.14)	
**At 12 months**	n = 29	n = 25	
Median (min–max)	1.12 (0.96 to 2.35)	1.21 (0.76 to 1.80)	0.340
Mean ± SD	1.19 ± 0.28	1.23 ± 0.26	
Change from start			
Median diff (min–max)	−0.33 (−0.82 to 0.68)	−0.32 (−1.15 to 0.30)	0.709
Mean diff (CI)	−0.33 (−0.45 to −0.21)	−0.32 (−0.46 to −0.17)	

* Significance level 0.05, Mann–Whitney U-test. ^a^ The site for the highest ratio at start was followed. Women were not included in analysis after the time-point when LRV exceeded ≥ 10%.

## Data Availability

The data presented in this study are available on request from the corresponding author. The data are not publicly available due to ethical reasons.

## References

[1] Chen K., Beeraka N.M., Zhang J., Reshetov I.V., Nikolenko V.N., Sinelnikov M.Y. (2021). Efficacy of da Vinci robot-assisted lymph node surgery than conventional axillary lymph node dissection in breast cancer—A comparative study. Int. J. Med. Robot..

[2] DiSipio T., Rye S., Newman B., Hayes S. (2013). Incidence of unilateral arm lymphoedema after breast cancer: A systematic review and meta-analysis. Lancet Oncol..

[3] Stanton A.W., Modi S., Mellor R.H., Levick J.R., Mortimer P.S. (2009). Recent advances in breast cancer-related lymphedema of the arm: Lymphatic pump failure and predisposing factors. Lymphat. Res. Biol..

[4] Johansson K., Branje E. (2010). Arm lymphoedema in a cohort of breast cancer survivors 10 years after diagnosis. Acta Oncol..

[5] Kilgore L.J., Korentager S.S., Hangge A.N., Amin A.L., Balanoff C.R., Larson K.E., Mitchell M.P., Chen J.G., Burgen E., Khan Q.J. (2018). Reducing Breast Cancer-Related Lymphedema (BCRL) Through Prospective Surveillance Monitoring Using Bioimpedance Spectroscopy (BIS) and Patient Directed Self-Interventions. Ann. Surg. Oncol..

[6] McNeely M.L., Peddle C.J., Yurick J.L., Dayes I.S., Mackey J.R. (2011). Conservative and dietary interventions for cancer-related lymphedema: A systematic review and meta-analysis. Cancer.

[7] Casley-Smith J. (1995). Alterations of untreated lymphedema and it’s grades over time. Lymphology.

[8] Bar Ad V., Cheville A., Solin L.J., Dutta P., Both S., Harris E.E. (2010). Time course of mild arm lymphedema after breast conservation treatment for early-stage breast cancer. Int. J. Radiat. Oncol. Biol. Phys..

[9] Soran A., Ozmen T., McGuire K.P., Diego E.J., McAuliffe P.F., Bonaventura M., Ahrendt G.M., DeGore L., Johnson R. (2014). The importance of detection of subclinical lymphedema for the prevention of breast cancer-related clinical lymphedema after axillary lymph node dissection; a prospective observational study. Lymphat. Res. Biol..

[10] Kaufman D.I., Shah C., Vicini F.A., Rizzi M. (2017). Utilization of bioimpedance spectroscopy in the prevention of chronic breast cancer-related lymphedema. Breast Cancer Res. Treat..

[11] Bundred N.J., Barrett E., Todd C., Morris J., Watterson D., Purushotham A., Riches K., Evans A., Skene A., Keeley V. (2023). Prevention of lymphoedema after axillary clearance by external compression sleeves PLACE randomised trial results. Effects of high BMI. Cancer Med..

[12] Ramos S., O´Donnell L., Knight G. (1999). Edema volume, not timing, is the key to success in lymphedema treatment. Am. J. Surg..

[13] Bundred N., Foden P., Todd C., Morris J., Watterson D., Purushotham A., Bramley M., Riches K., Hodgkiss T., Evans A. (2020). Increases in arm volume predict lymphoedema and quality of life deficits after axillary surgery: A prospective cohort study. Br. J. Cancer.

[14] Mazor M., Smoot B.J., Mastick J., Mausisa G., Paul S.M., Kober K.M., Elboim C., Singh K., Conley Y.P., Mickevicius G. (2019). Assessment of local tissue water in the arms and trunk of breast cancer survivors with and without upper extremity lymphoedema. Clin. Physiol. Funct. Imaging.

[15] Stout N.L., Pfalzer L.A., Levy E., McGarvey C., Springer B., Gerber L.H., Soballe P. (2011). Segmental limb volume change as a predictor of the onset of lymphedema in women with early breast cancer. PM&R.

[16] Thomis S., Dams L., Fourneau I., De Vrieze T., Nevelsteen I., Neven P., Gebruers N., Devoogdt N. (2020). Correlation Between Clinical Assessment and Lymphofluoroscopy in Patients with Breast Cancer-Related Lymphedema: A Study of Concurrent Validity. Lymphat. Res. Biol..

[17] Suami H. (2020). Anatomical Theories of the Pathophysiology of Cancer-Related Lymphoedema. Cancers.

[18] Karlsson K., Nilsson-Wikmar L., Brogardh C., Johansson K. (2020). Palpation of Increased Skin and Subcutaneous Thickness, Tissue Dielectric Constant, and Water Displacement Method for Diagnosis of Early Mild Arm Lymphedema. Lymphat. Res. Biol..

[19] Blom K.Y., Johansson K.I., Nilsson-Wikmar L.B., Brogårdh C.B. (2022). Early intervention with compression garments prevents progression in mild breast cancer-related arm lymphedema: A randomized controlled trial. Acta Oncol..

[20] Johansson K., Ingvar C., Albertsson M., Ekdahl C. (2001). Arm lymphedema, shoulder mobility and muscle strength after breast cancer treatment-a prospective 2 year study. Adv. Physiother..

[21] Lahtinen T., Seppala J., Viren T., Johansson K. (2015). Experimental and Analytical Comparisons of Tissue Dielectric Constant (TDC) and Bioimpedance Spectroscopy (BIS) in Assessment of Early Arm Lymphedema in Breast Cancer Patients after Axillary Surgery and Radiotherapy. Lymphat. Res. Biol..

[22] Mayrovitz H.N., Weingrad D.N., Lopez L. (2015). Assessing localized skin-to-fat water in arms of women with breast cancer via tissue dielectric constant measurements in pre- and post-surgery patients. Ann. Surg. Oncol..

[23] Specht M.C., Miller C.L., Russell T.A., Horick N., Skolny M.N., O’Toole J.A., Jammallo L.S., Niemierko A., Sadek B.T., Shenouda M.N. (2013). Defining a threshold for intervention in breast cancer-related lymphedema: What level of arm volume increase predicts progression?. Breast Cancer Res. Treat..

[24] Mahamaneerat W.K., Shyu C.-R., Stewart B.R., Armer J.M. (2008). Breast cancer treatment, BMI, post-op swelling/lymphedema. J. Lymphoedema.

[25] Ibrahim E.M., Al-Homaidh A. (2011). Physical activity and survival after breast cancer diagnosis: Meta-analysis of published studies. Med. Oncol..

[26] Lahart I.M., Metsios G.S., Nevill A.M., Carmichael A.R. (2015). Physical activity, risk of death and recurrence in breast cancer survivors: A systematic review and meta-analysis of epidemiological studies. Acta Oncol..

[27] Johansson K., Ohlsson K., Ingvar C., Albertsson M. (2002). Factors associated with the development of arm lymphedema following breast cancer treatment: A match pair case-control study. Lymphology.

[28] Ridner S., Deng J., Fu M.R., Radina E., Thiadens S.R., Weiss J., Dietrich M.S., Cormier J.N., Tuppo C.M., Armer J.M. (2012). Symptom burden and infection occurrence among individuals with extremity lymphedema. Lymphology.

[29] Moayedi M., Davis K.D. (2013). Theories of pain: From specificity to gate control. J. Neurophysiol..

[30] Karges J., Mark B., Stikeleather S., Worrell T. (2003). Concurrent validity of upper extremity volume estimates: Comparison of calculated volume derived from girth measurements and water displacement volume. Phys. Ther..

[31] Hidding J.T., Viehoff P.B., Beurskens C.H., van Laarhoven H.W., Nijhuis-van der Sanden M.W., van der Wees P.J. (2016). Measurement properties of instruments for measuring of lymphedema:systematic review. Phys Ther..

[32] ISL (2020). The diagnosis and treatment of peripheral lymphedema: 2020 Consensus document of the International Society of Lymphology. Lymphology.

[33] Taylor R., Jayasinghe U., Koelmeyer L., Ung O.A., Boyages J. (2006). Reliability and Validity of arm volume measurements for assessment of lymphedema. Phys. Ther..

[34] Mayrovitz H.N., Weingrad D.N., Brlit F., Lopez L.B., Desfor R. (2015). Tissue dielectric constant (Water) as an index of localized arm skin water: Differences between measuring probes and genders. Lymphology.

[35] Mayrovitz H.N., Davey S., Shapiro E. (2009). Suitability of single tissue dielectric constant measurements to assess local tissue water in normal and lymphedematous skin. Clin. Physiol. Funct. Imaging.

[36] Mayrovitz H.N., Weingrad D.N., Davey S. (2009). Local tissue water in at-risk and contralateral forearms of women with and without breast cancer treatment related lymphedema. Lymphat. Res. Biol..

[37] Frändin K., Grimby G. (1994). Assessment of physical activity, fitness and performance in 76 year olds. Scand. J. Med. Sci. Sport.

[38] Akita S., Nakamura R., Yamamoto N., Tokumoto H., Ishigaki T., Yamaji Y., Yoshitaro S., Yoshitaka K., Nobuyuki M., Kaneshige S. (2016). Early Detection of Lymphatic Disorder and Treatment for Lymphedema following Breast Cancer. Plast. Reconstr. Surg..

[39] Martin-Almedina S., Mortimer P.S., Ostergaard P. (2021). Development and physiological functions of the lymphatic system: Insights from human genetic studies of primary lymphedema. Physiol. Rev..

[40] Kilbreath S.L., Lee M.J., Refshauge K.M., Beith J.M., Ward L.C., Simpson J.M., Black D. (2013). Transient swelling versus lymphoedema in the first year following surgery for breast cancer. Support. Care Cancer.

[41] Stout Gergich N.L., Pfalzer L.A., McGarvey C., Springer B., Gerber L.H., Soballe P. (2008). Preoperative assessment enables the early diagnosis and successful treatment of lymphedema. Cancer.

[42] Bernas M., Witte C., Belch D., Summers P. (1996). Limb volume measurments in lymphedema issues and standards. Lymphology.

[43] Jonsson C., Johansson K., Bjurberg M., Brogardh C. (2022). Circumferential Measurements to Calculate Lower Limb Volume in Persons with Lymphedema: What Segment Length Is to Be Recommended?. Lymphat. Res. Biol..

[44] Hayes S.C., Singh B., Reul-Hirche H., Bloomquist K., Johansson K., Jonsson C., Plinsinga M. (2022). The Effect of Exercise for the Prevention and Treatment of Cancer-Related Lymphedema: A Systematic Review with Meta-analysis. Med. Sci. Sport. Exerc..

[45] Huang T., Tseng S., Lin C., Bai C., Chen C., Hung C., Huang C., Wu C., Tam K. (2013). Effects of manual lymphatic drainage on breast cancer-related lymphedema: A systematic review and meta-analysis of randomized controlled trial. World J. Surg. Oncol..

[46] Liang M., Chen Q., Peng K., Deng L., He L., Hou Y., Zhang Y., Guo J., Mei Z., Li Z. (2020). Manual lymphatic drainage for lymphedema in patients after breast cancer surgery: A systematic review and meta-analysis of randomized controlled trials. Medicine.

[47] De Vrieze T., Gebruers N., Nevelsteen I., Fieuws S., Thomis S., De Groef A., Tjalma W.A., Belgrado J.-P., Vandermeeren L., Monten C. (2022). Manual lymphatic drainage with or without fluoroscopy guidance did not substantially improve the effect of decongestive lymphatic therapy in people with breast cancer-related lymphoedema (EFforT-BCRL trial): A multicentre randomised trial. J. Physiother..

[48] Lymphoedema Framework Best Practice for the Management of Lymphoedema. International Consensus London: MEP Ltd., BP PAGESfinjune8 bQ5. lympho.org.

[49] Karlsson K., Biguet G., Johansson K., Nilsson-Wikmar L. (2015). Perceptions of lymphoedema treatment in patients with breast cancer—A patient perspective. Scand. J. Caring Sci..

[50] Zaleska M.T., Olszewski W.L. (2018). Indocyanine green near-infrared lymphangiography for evaluation of effectiveness of edema fluid flow under therapeutic compression. J. Biophotonics.

[51] Suami H., Koelmeyer L., Mackie H., Boyages J. (2018). Patterns of lymphatic drainage after axillary node dissection impact arm lymphoedema severity: A review of animal and clinical imaging studies. Surg. Oncol..

